# Genotypic Characterization of Clinical *Klebsiella* spp. Isolates Collected From Patients With Suspected Community-Onset Sepsis, Sweden

**DOI:** 10.3389/fmicb.2021.640408

**Published:** 2021-04-30

**Authors:** Patricia Saxenborn, John Baxter, Andreas Tilevik, Magnus Fagerlind, Fredrik Dyrkell, Anna-Karin Pernestig, Helena Enroth, Diana Tilevik

**Affiliations:** ^1^Systems Biology Research Centre, School of Bioscience, University of Skövde, Skövde, Sweden; ^2^1928 Diagnostics, Gothenburg, Sweden; ^3^Molecular Microbiology, Laboratory Medicine, Unilabs AB, Skövde, Sweden

**Keywords:** *Klebsiella*, whole-genome sequencing, antimicrobial susceptibility, clinical microbiology, multidrug resistance, nanopore-based sequencing, Illumina sequencing

## Abstract

*Klebsiella* is a genus of Gram-negative bacteria known to be opportunistic pathogens that may cause a variety of infections in humans. Highly drug-resistant *Klebsiella* species, especially *K. pneumoniae*, have emerged rapidly and are becoming a major concern in clinical management. Although *K. pneumoniae* is considered the most important pathogen within the genus, the true clinical significance of the other species is likely underrecognized due to the inability of conventional microbiological methods to distinguish between the species leading to high rates of misidentification. Bacterial whole-genome sequencing (WGS) enables precise species identification and characterization that other technologies do not allow. Herein, we have characterized the diversity and traits of *Klebsiella* spp. in community-onset infections by WGS of clinical isolates (*n* = 105) collected during a prospective sepsis study in Sweden. The sequencing revealed that 32 of the 82 isolates (39.0%) initially identified as *K. pneumoniae* with routine microbiological methods based on cultures followed by matrix-assisted laser desorption-time of flight mass spectrometry (MALDI-TOF MS) had been misidentified. Of these, 23 were identified as *Klebsiella variicola* and nine as other members of the *K. pneumoniae* complex. Comparisons of the number of resistance genes showed that significantly fewer resistance genes were detected in *Klebsiella oxytoca* compared to *K. pneumoniae* and *K. variicola* (both values of *p* < 0.001). Moreover, a high proportion of the isolates within the *K. pneumoniae* complex were predicted to be genotypically multidrug-resistant (MDR; 79/84, 94.0%) in contrast to *K. oxytoca* (3/16, 18.8%) and *Klebsiella michiganensis* (0/4, 0.0%). All isolates predicted as genotypically MDR were found to harbor the combination of β-lactam, fosfomycin, and quinolone resistance markers. Multi-locus sequence typing (MLST) revealed a high diversity of sequence types among the *Klebsiella* spp. with ST14 (10.0%) and ST5429 (10.0%) as the most prevalent ones for *K. pneumoniae*, ST146 for *K. variicola* (12.0%), and ST176 for *K. oxytoca* (25.0%). In conclusion, the results from this study highlight the importance of using high-resolution genotypic methods for identification and characterization of clinical *Klebsiella* spp. isolates. Our findings indicate that infections caused by other members of the *K. pneumoniae* complex than *K. pneumoniae* are a more common clinical problem than previously described, mainly due to high rates of misidentifications.

## Introduction

The Gram-negative bacteria *Klebsiella* possess a major threat to public health, causing significant morbidity and mortality worldwide. It is known to be an opportunistic pathogen that can cause a variety of health-care associated and community-acquired infections, such as urinary tract infections, pneumonia, sepsis, meningitis, and pyogenic liver abscesses. The emergence of highly drug-resistant strains as well as the increasing diversity of antibiotic resistance phenotypes observed in *Klebsiella* spp. are particularly concerning (Jean et al., 2018; Bengoechea and Sa Pessoa, 2019; Heinz et al., 2019; Rodríguez-Medina et al., 2019). *Klebsiella* infections in humans are caused mainly by *Klebsiella pneumoniae*, and to a lesser degree by *Klebsiella oxytoca* (Singh et al., 2016).

Recently, many related *Klebsiella* spp. have been identified and classified leading to an expansion of the taxonomy of the *K. pneumoniae* complex. *Klebsiella pneumoniae* has formerly been divided into three phylogroups (KpI, KpII, and KpIII), later classified as distinct species (*K. pneumoniae*, *Klebsiella quasipneumoniae*, and *Klebsiella variicola*; Brisse and Verhoef, 2001; Brisse et al., 2004, 2014; Rosenblueth et al., 2004). The *K. pneumoniae* complex currently comprises seven *K. pneumoniae*-related species: *K. pneumoniae*, *K. quasipneumoniae* subsp. *quasipneumoniae*, *K. quasipneumoniae* subsp. *similipneumoniae*, *K. variicola* subsp. *variicola* (herein referred to as *K. variicola*), *K. variicola* subsp. *tropicalensis*, *K. africanensis*, and the recently described *K. quasivariicola* (Long et al., 2017a). Although *K. pneumoniae* is the major cause of infections within the complex, the involvement in human infections of the other species is gaining recognition (Seki et al., 2013; Brisse et al., 2014; Maatallah et al., 2014; Holt et al., 2015; Breurec et al., 2016; Chen et al., 2016; Ejaz et al., 2017; Long et al., 2017b; Nicolás et al., 2018). Unfortunately, the inability of conventional microbiological methods to distinguish species within the complex results in high rates of misidentifications (Long et al., 2017b), thus masking the true clinical significance of each phylogroup and their epidemiological features (Seki et al., 2013; Brisse et al., 2014; Long et al., 2017b). Usually, members of the *K. pneumoniae* complex are misidentified as *K. pneumoniae* (Rodríguez-Medina et al., 2019). Nowadays, matrix-assisted laser desorption-time of flight mass spectrometry (MALDI-TOF MS) is commonly used for species identification within standard diagnostic routine in clinical laboratories but its ability to discriminate between *Klebsiella* spp. is highly dependent on the database version used (Long et al., 2017b; Rodrigues et al., 2018). Also identification methods based on PCR have been developed but those are prone to errors or do not distinguish all phylogroups (Bialek-Davenet et al., 2014; Brisse et al., 2014; Garza-Ramos et al., 2015). Reliable identification of the different members of the *K. pneumoniae* complex can thus only be achieved using whole-genome sequencing (WGS; Long et al., 2017b) or sequencing of specific genetic markers (e.g., *bla_LEN_*, *bla_OKP_*, *bla_SHV_*, *rpoB*, *gyrA*, and *parC*; Brisse et al., 2014; Holt et al., 2015).

In addition to accurate species identification, WGS-based analysis has rendered applicable for other aspects of clinical microbiology, including genotypic resistance prediction, virulence characterization, infectious disease control, and epidemiology of pathogens (Deurenberg et al., 2017; Tagini and Greub, 2017; Rossen et al., 2018). Thereof, WGS has been perceived as one of the most promising techniques in clinical microbiology (Bertelli and Greub, 2013; Deurenberg et al., 2017) and is today widely implemented in research laboratories. The implementation of bacterial WGS in the clinical laboratory has hitherto been hampered by long turn-around time, high cost, and the need for bioinformatics expertise. However, the recent development of long-read sequencers with relatively short runtimes as well as automated user-friendly bioinformatics pipelines may imply means to address these challenges (Petersen et al., 2019). The aim of this study was to characterize the diversity and traits of clinical *Klebsiella* spp. by WGS-based analysis using bioinformatics tools, and also to compare these results with those obtained by routine microbiological methods. A total of 105 *Klebsiella* spp. isolates, collected during a prospective, consecutive study in south-west Sweden of adult patients with suspected community-onset sepsis (Ljungström et al., 2019), were included.

## Materials and Methods

### Bacterial Isolates

From September 2011 to June 2012, a prospective observational study of community-onset severe sepsis and septic shock in adults was conducted at Skaraborg Hospital, a secondary hospital with 640 beds, in the western region of Sweden (Ljungström et al., 2019). All patients ≥18 years consecutively admitted to the emergency department for suspicion of community-onset sepsis were asked to participate in the study. The study was approved by the Regional Ethical Review Board of Gothenburg (376-11). As the present study only focused on bacterial isolates recovered from cultures included in the routine patient care, no individual written consent was needed. During 9 months, approximately 1,800 bacterial isolates from different sample types were recovered from the patients enrolled in the study. These isolates were cryopreserved at the time of recovery by transferring colonial material to Microbank™ vials (Pro-Lab Diagnostics, Ontario, Canada) stored at −80°C. For the present study, all isolates identified as *Klebsiella* spp. (*n* = 105) with routine microbiological methods based on cultures followed by MALDI-TOF MS (DB-4110) were selected. The isolates were recovered from 82 patients in samples collected from blood (*n* = 29), urine (*n* = 71), nasopharynx (*n* = 4), and wound (*n* = 1).

### MALDI-TOF MS Identification

Species identification of isolates was performed with MALDI-TOF MS in the clinical laboratory Unilabs at Skaraborg Hospital on a MicroFlex LT mass spectrometer (Bruker Daltonics, Germany) with BioTyper software v2.0 using default parameter settings as part of the routine clinical practice as described elsewhere (Ljungstrom et al., 2015; Enroth et al., 2019). Spectral scores above 2.0 were used as cut-off for correct identification. At the time of the study, the Bruker microorganism database MBT Compass Library DB-4110 (Bruker Daltonics, Germany) released in April 2011 was used.

### Phenotypic Antibiotic Susceptibility Testing

Antibiotic susceptibilities were determined by accredited laboratory methods using the disc diffusion method on Mueller-Hinton media according to European Committee on Antimicrobial Susceptibility Testing (EUCAST) guidelines.^1^ As the phenotypic antibiotic susceptibility testing (AST) was performed as part of the routine clinical practice, the sample type mainly determined which antibiotics to be tested for each bacterial isolate (Table 1).

**Table 1:** Specification of antibiotics used in the phenotypic antibiotic susceptibility testing (AST) for *Klebsiella* spp. isolates as part of the routine clinical practice.^1^

Antibiotic	Sample type			
	Blood	Nasopharynx	Urine	Wound
**Aminoglycoside**				
Tobramycin	●	●		●
**β-lactam**				
Ampicillin			●	
Cefadroxil	●	●	●	●
Ceftazidime	●			
Ceftibuten	●	●	●	●
Cefotaxime	●	●	●	●
Isoxazolyl penicillin	●			
Mecillinam			●	
Meropenem	●			
Piperacillin/tazobactam	●			●
**Chloramphenicol**				
Chloramphenicol			●	
**Nitrofuran**				
Nitrofurantoin	●			
**Quinolone**				
Ciprofloxacin	●	●	●	●
**Trimethoprim**				
Trimethoprim			●	
**Sulphonamide**				
Trimethoprim/sulphonamide	●	●	●	●

AST, antibiotic susceptibility testing; ESBL, extended spectrum β-lactam.

1 Supplementary resistance testing for additional antibiotics was performed when needed, e.g., upon suspicion of ESBL resistance.

### Illumina Dye Sequencing and Analysis of Short-Read Data

Genomic DNA was extracted from pure cultures using the MagNA Pure 96 DNA and Viral NA Small Volume Kit (Roche Diagnostics, Switzerland) on a MagNA Pure 96 instrument according to the Pathogen Universal 200 protocol (Roche Diagnostics, Switzerland). NexteraXT libraries were prepared using the manufacturer’s protocol (Illumina, San Diego, CA) and sequenced on an Illumina HiSeq instrument at SciLifeLab, Solna, Sweden.

Primary quality control of the FASTQ files was performed using the FastQC software (v.0.11.5) (Andrews, 2010). Trimmomatic (v.0.36) was used for adapter removal and quality trimming with a sliding window of size 4 and a minimum quality of 20 (Bolger et al., 2014). In addition, the first 12 bases were trimmed by the HEADCROP argument, and reads with a length shorter than 30 bp were removed. FASTQ files were then assembled into contigs using the St. Petersburg genome assembler (SPAdes v.3.11.1; Bankevich et al., 2012). The Quality Assessment Tool for Genome Assemblies (QUAST v.4.6.0) was used to assess the quality of the assembled contigs (Gurevich et al., 2013). Evaluation of the contigs in QUAST was performed using default settings with reference sequences obtained from the National Center for Biotechnology Information (NCBI). The reference sequence for *K. pneumoniae* subsp. *pneumoniae* HS11286 (GenBank accession number NC_016845.1) was used for *K. pneumoniae* and *K. variicola* isolates, and the reference sequence *K. oxytoca* CAV1374 (GenBank accession number NZ_CP011636.1) for *K. oxytoca* isolates (Gurevich et al., 2013). Species identification was performed by calculating the pairwise average nucleotide identity (ANI) based on BLAST+ (ANIb) in JSpeciesWS (Richter and Rosselló-Móra, 2009). The reference genomes for each bacterial species analyzed using ANI were *K. pneumoniae* HS11286 (GenBank accession number NC_016845.1), *K. quasipneumoniae* subsp. *quasipneumoniae* 01A030 (GenBank accession number NZ_CCDF01000032.1), *K. quasipneumoniae* subsp. *similipneumoniae* 07A044 (GenBank accession number NZ_CBZR010000026.1), *K. variicola* At-22 (GenBank accession number NC_013850.1), *K. quasivariicola* KPN1705 (GenBank accession number NZ_CP022823.1), *K. oxytoca* CAV1335 (GenBank accession number NZ_CP011618.1), *Klebsiella michiganensis* M1 (GenBank accession number NZ_CP008841.1), and *Escherichia coli* ATCC 25922 (GenBank accession number NC_000913.3). An ANI threshold of 96% or greater was considered to delineate species boundaries as a threshold of 96% correlates well to DNA-DNA hybridization (Richter and Rosselló-Móra, 2009; Ciufo et al., 2018).

The presence of plasmid replicons and acquired antibiotic resistance genes, including both chromosomal and plasmid-borne, was detected using free web-based tools hosted by the Center for Genomic Epidemiology (CGE) website^2^ including PlasmidFinder v.1.3 and ResFinder v.3.0. The tool PlasmidFinder was used with “*Enterobacteriaceae*” as selected database with default settings for threshold ID (95%) and minimum length (60%; Carattoli et al., 2014). The ResFinder database for detection of acquired antibiotic resistance genes in *Klebsiella* spp. included markers for 15 antibiotic classes (Table 2), and was used with default settings for threshold ID (90%) and minimum length (60%; Zankari et al., 2012). The presence of resistance genes was further investigated using the commercial cloud-based platform 1928^3^ (1928 Diagnostics, Sweden). The 1928 platform supports a more comprehensive analysis of *K. pneumoniae* than ResFinder by including markers for additional antibiotic classes (*n* = 26, Table 2). The FASTQ files for isolates initially reported as *K. pneumoniae* by MALDI-TOF MS (DB-4110) were uploaded for inferred antibiotic susceptibility based on genotype resistance markers (hereby and later referred to as predicted antibiotic susceptibility). On upload, the average sequence coverage was estimated. A coverage ≥30 was required for analysis to continue. The read data were queried for identifying genes from the genotype resistance database using an assembly-free method based on k-mer counting.

**Table 2:** Specification of antibiotic classes for which resistance markers were included in respective database.

Antibiotic class	ResFinder	1928
Aminoglycoside	●	●
β-lactam^1^	●	●
Broad-spectrum β-lactam		●
Carbapenemase		●
Chloramphenicol	●	●
Colistin	●	●
ESBL		●
Evernimicin		●
Fosfomycin	●	●
Fusidic acid	●	●
Glycopeptide antibiotics	●	●
MLS	●	●
Multidrug efflux pump		●
Mupirocin		●
Narrow-spectrum β-lactam		●
Nitroimidazole	●	●
Oxazolidinone	●	●
Pleuromutilin		●
Quinolone	●	●
Rifamycin	●	●
Sulphonamide	●	●
Tetracenomycin		●
Tetracycline	●	●
Thiopeptide		●
Trimethoprim	●	●
Viomycin		●

ESBL, extended spectrum β-lactam; MLS, macrolide, lincosamide, and streptogramin.

1 The predicted susceptibility for β-lactam provided by ResFinder covered the subclasses β-lactam, broad-spectrum β-lactam, and ESBL.

Multi-locus sequence typing (MLST) analysis was performed using free web-based MLST tools. For isolates genotypically identified as *K. michiganensis*, *K. oxytoca*, *K. pneumoniae*, *K. quasipneumoniae* subsp. *quasipneumoniae*, *K. quasipneumoniae* subsp. *similipneumoniae*, and *K. quasivariicola*, the MLST tool v.2.0 hosted by the CGE website^4^ was used. This MLST scheme consists of alleles from the following seven loci: *gapA*, *infB*, *mdh*, *pgi*, *phoE*, *rpoB*, and *tonB*. The selected MLST configuration for isolates identified as *K. pneumoniae*, *K. quasipneumoniae* subsp. *quasipneumoniae*, *K. quasipneumoniae* subsp. *similipneumoniae*, or *K. quasivariicola* was “*K. pneumoniae*,” and “*K. oxytoca*” for the *K. oxytoca* and *K. michiganensis* isolates (Larsen et al., 2012). For one of the *K. oxytoca* isolates (KLO411), the sequence type (ST) profile could not be determined. Although exact matches for all seven loci were found, they did not match any known ST profile and the sequence data did not meet the quality requirements at the PubMLST website^5^ to assign a new ST profile. For the isolates genotypically identified as *K. variicola*, MLST was performed using the online service “MLST *K. variicola*” hosted by Instituto Nacional de Salud Pública.^6^ This MLST scheme consists of alleles from the following seven loci: *leuS*, *pgi*, *pgk*, *phoE*, *pyrG*, *rpoB*, and *fusA* (Barrios-Camacho et al., 2019). For one of the *K. variicola* isolates (KLP1399), the ST profile could not be determined as the nucleotide sequence for the *phoE* gene was incomplete.

### Nanopore-Based Sequencing and Analysis of Long-Read Data

As a pilot validation study, 12 of the *Klebsiella* spp. isolates were also subject to nanopore-based sequencing using a MinION instrument (Oxford Nanopore Technology, United Kingdom). The number of isolates to include was set by the number of samples that could be sequenced simultaneously on the MinION device. Six of the isolates were selected based on the high quality of existing Illumina short-read data. The remaining six isolates were chosen due to inconsistent results for species identification with MALDI-TOF MS (DB-4110) compared to the genotypic analysis of Illumina short-read data. Species identification by MALDI-TOF MS was reanalyzed for these 12 isolates in 2019 using the Bruker microorganism database MBT Compass Library DB-7854 (Bruker Daltonics, Germany) released in April 2018.

For extraction of bacterial DNA for nanopore sequencing, the QIAGEN Genomic-tip 20/G 20/G (QIAGEN, Germany) was used according to the manufacturer’s instructions. Purified DNA was then prepared for nanopore sequencing using the Rapid Barcoding kit SQK-RBK004 (Oxford Nanopore Technologies, United Kingdom) following the manufacturer’s protocol. Sequencing was performed on MinION flow cells (R.1.9 FLO-MIN-106, Oxford Nanopore Technologies, United Kingdom) and data collected using the MinKNOW software v2.0. Automated analysis of the FASTQ files was performed with the cloud-based platform EPI2ME (Oxford Nanopore Technologies, United Kingdom) using What’s-In-My-Pot (WIMP), a Centrifuge-based system for species identification, and Antimicrobial Resistance Mapping Application (ARMA) for detection of antibiotic resistance genes.

### Statistical Analysis

Statistical analyses were performed using R v.4.0.3 (R Foundation for Statistical Computing, Austria). All tests were two-sided, and *p* < 0.05 was considered statistically significant. Poisson and quasi-Poisson regression analysis was performed for comparisons of count data, and adjustment of *p*-values for multiple comparisons was made using the Holm method. Dispersion test was performed using the R package AER v.1.2-9. Concordances between conventional microbiological methods and bioinformatics analysis tools were tested with Cohen’s kappa statistics for inter-rater agreement using cut-off values for the kappa value as described elsewhere (Landis and Koch, 1977). The kappa values were computed using the R package irr v.0.84.1. For the construction of 95% CI for kappa values, the basic bootstrapping method in the R package boot v.1.3-24 was used. The number of bootstrapping was 1,000. For the construction of 95% CI for proportions of agreement between methods, the Agresti-Coull method was used (Agresti and Coull, 1998). Figures were constructed using R v.4.0.3 (R Foundation for Statistical Computing, Austria) and the data visualization R packages ggplot2 v.3.2.1 and ggsci v.2.9.

## Results

### Misidentification of *Klebsiella* spp. by MALDI-TOF MS

**Figure 1:**
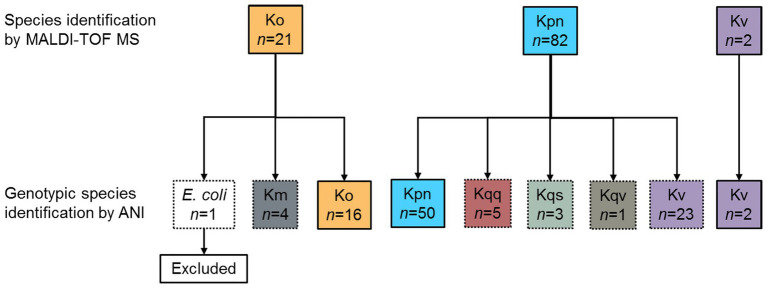
Overview of the results of the species identification. During the prospective sepsis study, species identification by MALDI-TOF MS (DB-4110) was performed for all collected bacterial isolates as part of the routine clinical practices. All isolates initially identified as *Klebsiella* spp. (*n* = 105) were subject to whole-genome sequencing (WGS). Genotypic species identification was performed by calculating the pairwise ANI on the assembled Illumina short-read data against reference genomes. The species identification by MALDI-TOF MS (DB-4110) and ANI agreed (solid line boxes) for 68 isolates, whereas the species were corrected based on the ANI results (dotted line boxes) for the remaining 37 isolates. ANI, average nucleotide identity; Km, *K. michiganensis*; Ko, *K. oxytoca*; Kpn, *K. pneumoniae*; Kqq, *K. quasipneumoniae* subsp. *quasipneumoniae*; Kqs, *K. quasipneumoniae* subsp. *similipneumoniae*; and Kqv, *K. quasivariicola*, Kv *K. variicola*.

During the prospective sepsis study, species identification by MALDI-TOF MS (DB-4110) was performed for all collected bacterial isolates as part of the routine clinical practice. A total of 105 bacterial isolates were identified as *Klebsiella* spp., whereof 82 isolates were identified as *K. pneumoniae*, two isolates as *K. variicola*, and 21 isolates as *K. oxytoca* (Figure 1). Due to previous reports pointing out the inability of conventional microbiology laboratory techniques to differentiate between *Klebsiella* spp. (Long et al., 2017a; Rodríguez-Medina et al., 2019), genotypic species identification of the collected isolates was performed by calculating the pairwise ANI on the assembled Illumina short-read data against reference genomes. Out of the 82 isolates initially reported as *K. pneumoniae*, the species were corrected for 32 (39.0%, 95% CI 29.2–49.9) based on the results from the genotypic species identification. Of these, 23 were genotypically identified as *K. variicola*, five as *K. quasipneumoniae* subsp. *quasipneumoniae*, three as *K. quasipneumoniae* subsp. *similipneumoniae*, and one as *K. quasivariicola*. For both isolates initially reported as *K. variicola* by MALDI-TOF MS (DB-4110), the genotypic species identification was in agreement. For the 21 isolates initially reported as *K. oxytoca*, 16 were genotypically identified as *K. oxytoca*, four as *K. michiganensis*, and one as *E. coli*. The isolate identified as *E. coli* was excluded from further analysis as the current study focuses on *Klebsiella* spp. The species identification by MALDI-TOF MS (DB-4110) and ANI was discordant for 37.1% (37/105, 95% CI 26.8–44.8) of the isolates (Cohen’s kappa = 0.41, 95% CI 0.28–0.54).

### Genotypic Antibiotic Susceptibility Prediction

**Figure 2:**
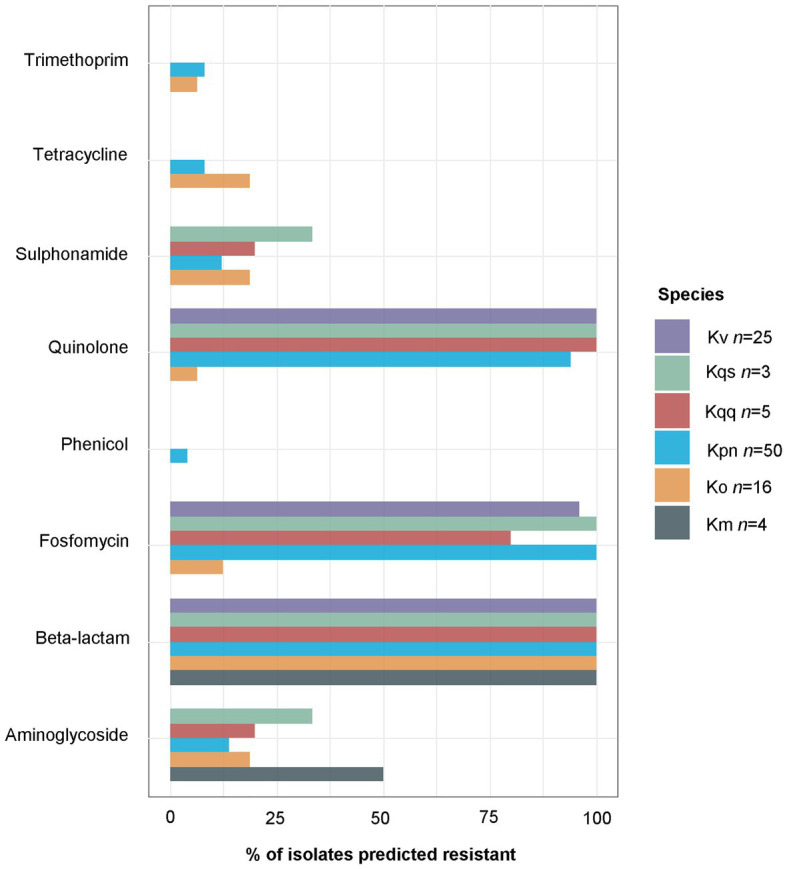
Percentages of isolates per species carrying at least one resistance marker against at least one agent included in the current antibiotic class as predicted by ResFinder. At least one gene conferring resistance against β-lactam antibiotics was predicted for all *Klebsiella* isolates, and at least one quinolone resistance gene was predicted for all *K. variicola*, *K. quasipneumoniae* subsp. *quasipneumoniae*, and *K. quasipneumoniae* subsp. *similipneumoniae*. The single *K. quasivariicola* isolate was predicted resistant against β-lactam, fosfomycin, and quinolone, data not included in the figure. Km, *K. michiganensis*; Ko, *K. oxytoca*; Kpn, *K. pneumoniae*; Kqq, *K. quasipneumoniae* subsp. *quasipneumoniae*; Kqs, *K. quasipneumoniae* subsp. *simili*; and Kv *K. variicola*.

**Figure 3:**
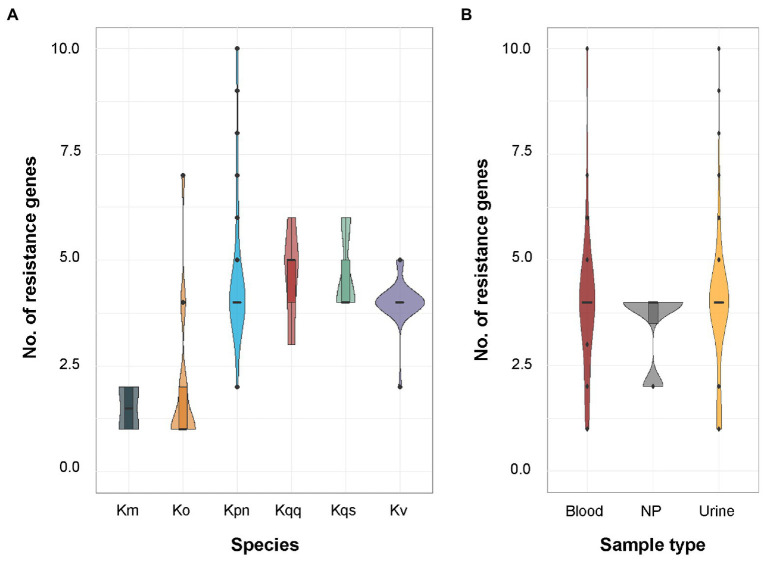
**(A)** Distribution of the number of resistance genes predicted by ResFinder per species. The sample size for each species was *K. oxytoca* (*n* = 16), *K. michiganensis* (*n* = 4), *K. pneumoniae* (*n* = 50), *K. quasipneumoniae* subsp. *quasipneumoniae* (*n* = 5), *K. quasipneumoniae* subsp. *similipneumoniae* (*n* = 3), *K. variicola* (*n* = 25), and *K. quasivariicola* (*n* = 1). Significant differences in the number of predicted resistance markers were observed between *K. pneumoniae* and *K. oxytoca* (*p* < 0.001), and between *K. oxytoca* and *K. variicola* (*p* < 0.001) using pairwise quasi-Poisson regression followed by adjustment of the resulting *p*-values by the Holm method. The single *K. quasivariicola* isolate was predicted resistant against β-lactam, fosfomycin, and quinolone, data not included in the figure. **(B)** Distribution of the number of resistance genes predicted by ResFinder per sample type. The sample size for each sample type was blood (*n* = 29), urine (*n* = 70), and nasopharynx (*n* = 4). No significant differences were detected between any sample types using pairwise Poisson regression (all values of *p* > 0.05). Km, *K. michiganensis*; Ko, *K. oxytoca*, Kpn, *K. pneumoniae*; Kqq, *K. quasipneumoniae* subsp. *quasipneumoniae*; Kqs, *K. quasipneumoniae* subsp. *similipneumoniae*; Kv, *K. variicola*; and NP, nasopharynx.

The tool ResFinder was used on all *Klebsiella* spp. isolates to detect the presence of acquired antibiotic resistance genes. ResFinder detected at least one β-lactam resistance gene in all *Klebsiella* spp. isolates (*n* = 104, Figure 2). The second most frequently predicted resistance was against fosfomycin (*n* = 84) followed by quinolone (*n* = 82). For *K. pneumoniae*, all isolates were predicted to be resistant against both β-lactam and fosfomycin, and 94.0% (47/50) to quinolone. A similar resistance profile was observed for *K. quasipneumoniae* subsp. *quasipneumoniae*, *K. quasipneumoniae* subsp. *similipneumoniae*, *K. variicola*, and *K. quasivariicola* with 100.0% of the isolates predicted to be resistant to β-lactam, quinolone, and fosfomycin. For *K. oxytoca* and *K. michiganensis*, the most commonly predicted resistance was against β-lactam antibiotics (100.0%, Figure 2). Beyond that, quite few resistance markers were detected among the *K. oxytoca* and *K. michiganensis* isolates. Comparison of the number of resistance genes showed that significantly fewer resistance genes were detected in *K. oxytoca* compared to *K. pneumoniae* and *K. variicola* (both values of *p* < 0.001, Figure 3A). Four resistance genes were detected by ResFinder in the single *K. quasivariicola* isolate, not included in the statistical comparison. No significant differences in the number of detected resistance genes were observed between sample types (all values of *p* > 0.05, Figure 3B). Seven resistance genes were detected by ResFinder in the only isolate collected from wound, not included in the statistical comparison.

To determine the agreement between phenotypic and genotypic resistance on Illumina short-reads, the results from the phenotypic AST and ResFinder were compared (Table 3). In 65.8% (231/351, 95% CI 60.7–70.6) of the cases, the phenotypic AST and the antibiotic susceptibility predicted by ResFinder agreed (Cohen’s kappa = 0.35, 95% CI 0.27–0.43). Not all isolates were tested for all antibiotics in clinical routine (Table 1), therefore, the number of phenotypic results for each antibiotic varied and were less than the number of resistance genotypes predicted by ResFinder.

**Table 3:** Comparisons of phenotypic AST and predicted antibiotic susceptibility by ResFinder.

Antibiotic	Phenotypic AST vs. predicted antibiotic susceptibility by ResFinder				Discordant across methods [*n* (%)]
	RR	SS	RS	SR	
Aminoglycoside (*n* = 49)^1^	0	42	0	7	7 (14.3)
β-lactam (*n* = 101)^2^	73	0	0	28	28 (27.7)
Fluoroquinolone (*n* = 103)^2^	0	22	0	81	81 (80.2)
Chloramphenicol (*n* = 27)	1	25	1	0	1 (3.7)
Trimethoprim (*n* = 71)	4	64	3	0	3 (4.2)
Total (% of 351)	78 (22.2)	153 (43.6)	4 (1.1)	116 (33.0)	120 (34.2)

AST, antibiotic susceptibility testing; RR resistant by both phenotypic AST and ResFinder; SS susceptible by both phenotypic AST and ResFinder; RS resistant by phenotypic AST and susceptible by ResFinder; SR susceptible by phenotypic AST and resistant by ResFinder; and EUCAST, European committee on antimicrobial susceptibility testing.

1 For additional six isolates, the results from EUCAST disc diffusion were susceptible/indeterminate and could therefore not be determined. These results are not included in the table. ResFinder did not detect any resistance genes to aminoglycoside in these isolates.

2 For additional one isolate, the results from EUCAST disc diffusion were susceptible/indeterminate and could therefore not be determined. This result is not included in the table. This isolate was predicted to be resistant by ResFinder.

The ResFinder results were also compared to the predicted antibiotic susceptibilities using the 1928 platform for the 82 isolates identified as *K. pneumoniae* by MALDI-TOF MS (DB-4110). It should be noted that 1928 provided information about β-lactam resistance divided into three subclasses: β-lactam, broad-spectrum β-lactam, and extended spectrum β-lactam (ESBL), whereas the output from ResFinder was only β-lactam thus covering all three sub-classes. To enable a fair comparison, the output data from 1928 were merged into one β-lactam group covering resistance markers detected in all three sub-classes. The agreement between 1928 and ResFinder was 100.0% for all antibiotics, except fosfomycin where 1928 detected the resistance marker *fosA* in two isolates predicted as susceptible by ResFinder (Table 4). Overall, 99.7% of the predicted antibiotic susceptibilities were identical between ResFinder and 1928, demonstrating high overall agreement (Cohen’s kappa = 0.99, 95% CI 0.99–1.00).

**Table 4:** Comparisons of predicted antibiotic susceptibility by ResFinder and 1928 for the 82 isolates initially reported as *K. pneumoniae* by matrix-assisted laser desorption-time of flight mass spectrometry (MALDI-TOF MS; DB-4110).

Antibiotic	Antibiotic susceptibility prediction by ResFinder and 1928				Discordant across methods [*n* (%)]
	RR	SS	RS	SR	
Aminoglycoside	10	72	0	0	0 (0)
β-lactam	82	0	0	0	0 (0)
Fluoroquinolone	79	3	0	0	0 (0)
Fosfomycin	80	0	0	2	2 (2.4)
Chloramphenicol	2	80	0	0	0 (0)
Sulphonamide	9	73	0	0	0 (0)
Tetracycline	4	78	0	0	0 (0)
Trimethoprim	4	78	0	0	0 (0)
Total (% of 656)^1^	270 (41.2)	384 (58.5)	0 (0.0)	2 (0.3)	2 (0.3)

RR resistant by both ResFinder and 1928; SS susceptible by both ResFinder and 1928; RS resistant by ResFinder and susceptible by 1928; and SR susceptible by ResFinder and resistant by 1928.

1 Eighty-two isolates have been examined for eight antibiotics generating 656 comparisons in total.

The predicted antibiotic susceptibilities by 1928 were also compared to the resistance phenotypes (Table 5). In 61.6% (173/281, 95% CI 55.8–67.1%) of the cases, the resistance phenotype and the predicted antibiotic susceptibility by 1928 agreed (Cohen’s kappa = 0.30, 95% CI 0.22–0.38). In 15 isolates, all genotypically identified as *K. pneumoniae*, an ESBL resistance gene was detected by the 1928 platform (10 *bla_SHV-1_*, 3 *bla_SHV-27_*, and 2 *bla_SHV-41_*), implying that 30.0% (15/50, 95% CI 19.1–43.9) of the *K. pneumoniae* isolates in this study is genotypically predicted to be ESBL.

**Table 5:** Comparisons of phenotypic AST and predicted antibiotic susceptibility by 1928.

Antibiotic	Phenotypic AST vs. predicted antibiotic susceptibility by 1928				Discordant across methods [*n* (%)]
	RR	SS	RS	SR	
Aminoglycoside (*n* = 41)^1^	0	37	0	4	4 (9.8)
β-lactam (*n* = 79)^2^	57	0	0	22	22 (27.8)
Fluoroquinolone (*n* = 81)^2^	0	3	0	78	78 (96.3)
Chloramphenicol (*n* = 24)^3^	1	22	1	0	1 (4.2)
Trimethoprim (*n* = 56)	4	49	3	0	3 (5.4)
Total (% of 281)	62 (22.1)	111 (39.5)	4 (1.4)	104 (37.0)	108 (38.4)

AST, antibiotic susceptibility testing; RR resistant by both phenotypic AST and 1928; SS susceptible by both phenotypic AST and 1928; RS resistant by phenotypic AST and susceptible by 1928; SR susceptible by phenotypic AST and resistant by 1928; and EUCAST, European committee on antimicrobial susceptibility testing.

1 For additional five strains, the results from EUCAST disc diffusion were susceptible/indeterminate and could therefore not be determined. These results are not included in the table. All five strains were predicted to be susceptible by 1928.

2 For additional one strain, the results from EUCAST disc diffusion were susceptible/indeterminate and could therefore not be determined. This result is not included in the table. This strain was predicted to be resistant by 1928.

3 For additional one strain, the results from EUCAST disc diffusion were susceptible/resistant and could therefore not be determined. This result is not included in the table. This strain was predicted to be susceptible by 1928.

By defining genotypically MDR as the detection of at least one resistance gene in at least three of the antibiotic classes included in ResFinder (Table 4), a total of 94.0% (79/84) of the isolates within the *K. pneumoniae* complex were predicted to be genotypically MDR. More specifically, high percentages of MDR were observed in *K. pneumoniae* (47/50, 94.0%), *K. variicola* (24/25, 96.0%), *K. quasipneumoniae* subsp. *quasipneumoniae* (4/5, 80.0%), and *K. quasipneumoniae* subsp. *similipneumoniae* (3/3, 100.0%) but not in *K. oxytoca* (3/16, 18.8%) and *K. michiganensis* (0/4, 0.0%). The single *K. quasivariicola* isolate was also found to be MDR. All MDR *K. pneumoniae*, *K. variicola*, *K. quasipneumoniae* subsp. *quasipneumoniae*, and *K. quasipneumoniae* subsp. *similipneumoniae* isolates carried the combination of β-lactam, fosfomycin, and quinolone resistance determinants.

### High Diversity of Sequence Types

**Figure 4:**
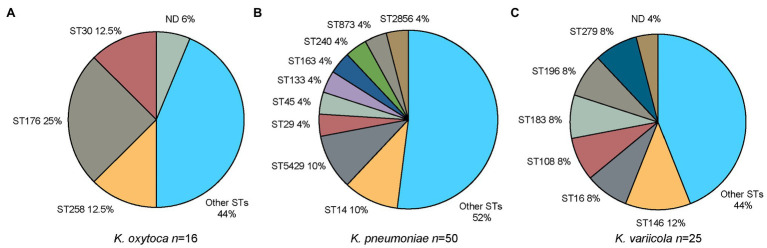
Distribution of sequence types (STs) for the three major groups of *Klebsiella* spp. in the current study. **(A)** *K. oxytoca*. “Other STs” include one isolate each of ST36, ST37, ST201, ST288, ST357, ST358, and ST359. “ND” refers to a single isolate for which the ST profile could not be determined. **(B)** *K. pneumoniae*. “Other STs” include one isolate each of ST11, ST20, ST30, ST37, ST39, ST187, ST215, ST220, ST294, ST309, ST381, ST485, ST462, ST678, ST685, ST788, ST870, ST872, ST966, ST1114, ST1948, ST3370, ST4069, ST4631, ST4725, and ST5442. **(C)** *K. variicola*. “Other STs” include one isolate each of ST149, ST261, ST280, ST281, ST282, ST283, ST284, ST285, ST286, ST287, and ST288. “ND” refers to a single isolate for which the ST profile could not be determined. MLST, multi-locus sequence typing; ND, not determined; and ST sequence type.

The typing technique MLST was used to further characterize the *Klebsiella* spp. isolates based on sequence differences in seven house-keeping genes. A high diversity of STs was detected among the *Klebsiella* spp. isolates. For *K. oxytoca*, 10 distinct STs were detected with ST176 being the most common one (4/16, 25.0%; Figure 4A), whereas a distinct ST was determined for each of the four *K. michiganensis* isolates (ST85, ST194, ST231, and ST356). Thirty-five distinct STs were identified for *K. pneumoniae* with ST14 (5/50, 10.0%; Figure 4B) and ST5429 being the most prevalent ones (5/50, 10.0%; Figure 4B). For the three *K. quasipneumoniae* subsp. *similipneumoniae* isolates, two were recognized as ST1647 and one as ST4768. For the five *K. quasipneumoniae* subsp. *quasipneumoniae* isolates, two were identified as ST5443, and one each of ST1887, ST4630, and ST5444. The single *K. quasivariicola* isolate was determined to be ST3497. For *K. variicola*, 17 distinct STs were identified with ST146 most common (3/25, 12.0%; Figure 4C).

### Plasmid Replicons

**Figure 5:**
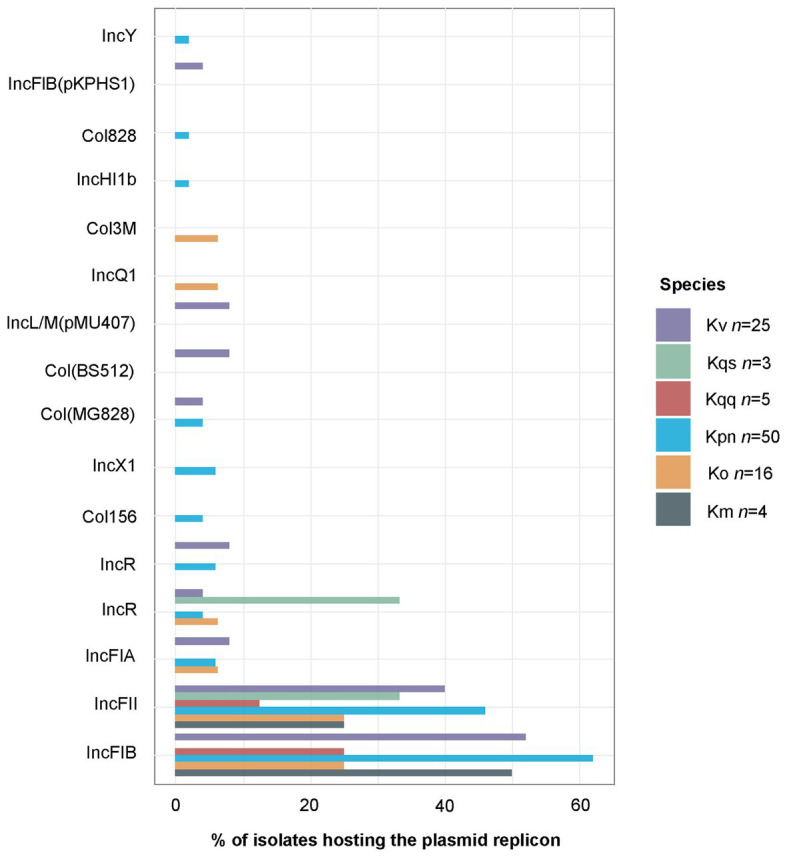
Percentage of isolates hosting each plasmid replicon type as predicted by PlasmidFinder. The total of 142 predicted plasmid replicons were distributed per species as follows: *K. michiganensis n* = 3, *K. oxytoca n* = 13, *K. pneumoniae n* = 84, *K. quasipneumoniae* subsp. quasipneumoniae *n*=3, *K. quasipneumoniae* subsp. *similipneumoniae n* = 2, *K. variicola n* = 36, and *K. quasivariicola n* = 1. The single *K. quasivariicola* isolate was predicted to host the plasmid replicon Col156, data not included in the figure. Km, *K. michiganensis*; Ko, *K. oxytoca*; Kpn, *K. pneumoniae*; Kqq, *K. quasipneumoniae* subsp. *quasipneumoniae*; Kqs, *K. quasipneumoniae* subsp. *similipneumoniae*; and Kv, *K. variicola*.

**Figure 6:**
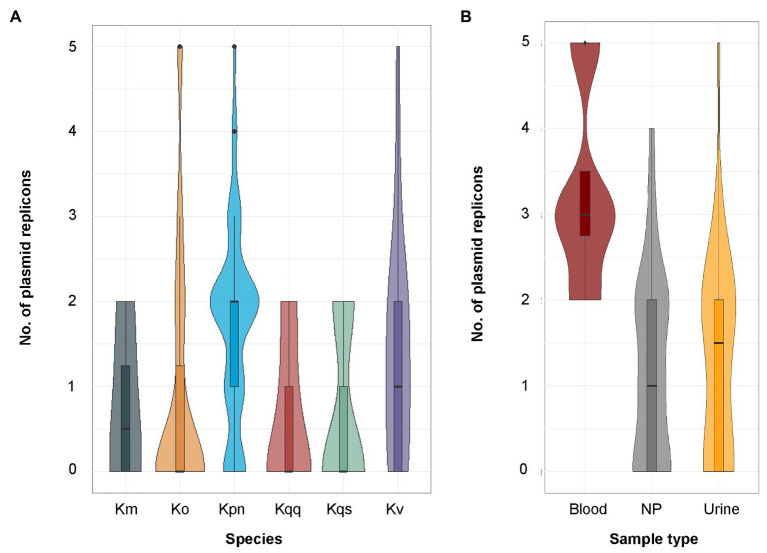
**(A)** Distribution of the number of plasmid replicons predicted by PlasmidFinder per species. The sample size for each species was *K. oxytoca* (*n* = 16), *K. michiganensis* (*n* = 4), *K. pneumoniae* (*n* = 50), *K. quasipneumoniae* subsp. *quasipneumoniae* (*n* = 5), *K. quasipneumoniae* subsp. *similipneumoniae* (*n* = 3), *K. variicola* (*n* = 25), and *K. quasivariicola* (*n* = 1, data not included in the figure). No significant differences in the number of predicted plasmid replicons were observed between any of the species using pairwise Poisson regression (all values of *p* > 0.05). **(B)** Distribution of the number of plasmid replicons predicted by PlasmidFinder per sample type. The sample size for each sample type was blood (*n* = 29), nasopharynx (*n* = 4), and urine (*n* = 70). Significant differences in the number of predicted plasmid replicons were observed between blood and nasopharynx (*p* = 0.01), and between urine and nasopharynx (*p* = 0.003), but not between blood and urine (*p* = 0.60) using pairwise Poisson regression followed by adjustment of the resulting *p*-values by the Holm method. Km, *K. michiganensis*; Ko, *K. oxytoca*; Kpn, *K. pneumoniae*; Kqq, *K. quasipneumoniae* subsp. *quasipneumoniae*; Kqs, *K. quasipneumoniae* subsp. *similipneumoniae*; Kv, *K. variicola*; and NP, nasopharynx.

In total, 16 different plasmid replicon types were detected by PlasmidFinder (Figure 5). The most common plasmid replicon types predicted were IncFIB, whereof 60 were found in 47 isolates, and IncFII, whereof 47 were found in 40 isolates. Three isolates (KLO1516 *K. oxytoca*, KLP307 *K. variicola*, and KLP1815 *K. pneumoniae*) were found to host five plasmid replicons. These isolates were collected from wound, nasopharynx, and blood, respectively. Additionally three isolates (KLP139 *K. pneumoniae*, KLP188 *K. pneumoniae*, and KLP189 *K. variicola*) collected from urine were detected to have four plasmid replicons. The number of plasmid replicons was compared between genotypic species and sample types. No significant differences in the number of plasmid replicons were observed between species (Figure 6A). Comparisons of the number of plasmid replicons between isolates from different sample types were performed for all sample types but wound due to too few isolates collected (*n* = 1). A significantly higher number of plasmid replicons was detected in isolates from nasopharynx in comparison to blood (*p* = 0.01), and urine (*p* = 0.003; Figure 6B).

### Nanopore-Based Sequencing

To evaluate long-reads for microbial profiling, 12 isolates were sequenced using the MinION device and also re-analyzed using an updated database version of MALDI-TOF MS (DB-7854). For only six of the isolates, the species identification using MALDI-TOF MS (DB-7854) matched the results of ANI (50.0%, 95% CI 25.5–74.5), whereas the species identification with WIMP on MinION long-read data was in agreement with ANI for nine isolates (75.0%, 95% CI 46.0–91.5; Table 6). All 12 isolates were predicted to be resistant to β-lactam antibiotics with ARMA on MinION long-read data consistent with ResFinder on Illumina short-read data. Interestingly, all 12 isolates were as well predicted to be resistant to colistin by ARMA, whereas no colistin resistance genes were detected by ResFinder. Otherwise, the antibiotic susceptibility prediction by ARMA and ResFinder found generally consistent matches.

**Table 6:** Comparisons of species identification by MALDI-TOF MS and genotypic species identification by bioinformatics tools.

Isolate	Species identification by MALDI-TOF MS		Genotypic species identification	
	Library DB-4110	Library DB-7854	Illumina short-reads^1^	MinION long-reads^2^
KLO25	*Klebsiella oxytoca*	*K. oxytoca*	*Klebsiella michiganensis*	*K. oxytoca*
KLO1508	*K. oxytoca*	*Klebsiella variicola/oxytoca*	*K. michiganensis*	*K. oxytoca*
KLO1695	*K. oxytoca*	*K. oxytoca*	*K. michiganensis*	*K. sp. LTGPAF-6F*
KLP205	*Klebsiella pneumoniae*	*K. pneumoniae*	*K. pneumoniae*	*K. pneumoniae*
KLP518	*K. pneumoniae*	*K. pneumoniae*	*K. pneumoniae*	*K. pneumoniae*
KLP918	*K. pneumoniae*	*K. variicola*	*K. variicola*	*K. variicola*
KLP1025	*K. pneumoniae*	*K. variicola*	*K. variicola*	*K. variicola*
KLP1293	*K. pneumoniae*	*K. pneumoniae*	*K. pneumoniae*	*K. pneumoniae*
KLP1442	*K. pneumoniae*	*K. pneumoniae*	*K. variicola*	*K. variicola*
KLP1818	*K. pneumoniae*	*K. pneumoniae*	*Klebsiella quasipneumoniae* subsp. *quasipneumoniae*	*K. quasipneumoniae*
KLP1935	*K. pneumoniae*	*K. variicola*	*K. variicola*	*K. variicola*
KLP2020	*K. pneumoniae*	*K. pneumoniae*	*K. quasipneumoniae* subsp. *quasipneumoniae*	*K. quasipneumoniae*

ANI, average nucleotide identity; MALDI-TOF MS, matrix-assisted laser desorption-time of flight mass spectrometry; and WIMP, What’s-In-My-Pot.

1 Species as determined by pairwise comparisons using ANI.

2 Majority species identification using WIMP.

## Discussion

In this study, we have characterized a collection of clinical *Klebsiella* spp. isolates using WGS-based analysis. Species identification based on Illumina short-read data was performed by calculating the pairwise ANI against reference genomes, and the results were compared to those obtained by routine microbiological methods. Among the 82 isolates identified as *K. pneumoniae* with MALDI-TOF MS (DB-4110), as many as 32 were genotypically identified as another *Klebsiella* spp. (39.0%, Figure 1) by ANI. This is in agreement with previous studies reporting that around 30% of clinical isolates initially identified as *K. pneumoniae* in clinical routine were later recognized as *K. variicola* or *K. quasipneumoniae* based on genotypic identification (Maatallah et al., 2014; Imai et al., 2019). The extensive misidentification of species within the *Klebsiella* complex has likely led to an underestimation of the clinical significance of other species than *K. pneumoniae*. A previous report from Stockholm, Sweden (Maatallah et al., 2014) suggests these organisms as a probable underrecognized cause of bacteremia, including fatal infections. Concerning the ability of MALDI-TOF MS to differentiate the species constituting the *K. pneumoniae* complex, it should be noted that recent updates of the Bruker reference database now should allow for identification of *K. variicola* from *K. pneumoniae* but not for the rest of the members of *K. pneumoniae* complex (Long et al., 2017a; Rodrigues et al., 2018). Our results for the 12 isolates re-analyzed with MALDI-TOF MS using an updated reference database (DB-7854) partially support this as three out of four *K. variicola* isolates then were correctly identified (Table 6). In addition to the misidentification of members of the *K. pneumoniae* complex, the species for five of 21 isolates initially reported as *K. oxytoca* with MALDI-TOF MS (DB-4110) were corrected when using ANI on the Illumina short-read data (Figure 1). Four isolates were genotypically identified as the closely related *K. michiganensis* as reported previously (Founou et al., 2019; Chapman et al., 2020), and one isolate as *E. coli*.

A correlation between β-lactamase genes and species has formerly been reported where *K. pneumoniae* is associated with *bla_SHV_*, *K. quasipneumoniae* with *bla_OKP_*, and *K. variicola* with *bla_LEN_* (Haeggman et al., 2004). This is consistent with our results from ResFinder since all *K. variicola*, as well as the *K. quasivariicola* isolate, harbored the *bla_LEN_* gene, all *K. quasipneumoniae* subsp. *quasipneumoniae* and *K. quasipneumoniae* subsp. *similipneumoniae* had the *bla_OKP_*, and all *K. pneumoniae* isolates carried the *bla_SHV_*. Multiplex PCR assays targeting specific core chromosomal β-lactamase genes have previously been proposed as potential methods for differentiation between the *Klebsiella* spp. (Fonseca et al., 2017). However, although there is an association between the above mentioned β-lactamase genes with the chromosomes of *K. pneumoniae*, *K. quasipneumoniae*, and *K. variicola*, several studies have reported findings contradicting the use of such lab-developed multiplex PCR methods for species identification. For example, the presence of the gene *bla_SHV_* on plasmids in isolates belonging to all three species has been revealed previously (Garza-Ramos et al., 2007; Long et al., 2017a). In addition, there are reports describing *K. variicola* isolates for which the chromosomal *bla_LEN_* gene has not been identified (Long et al., 2017a; Barrios-Camacho et al., 2019).

Although the emergence of highly drug-resistant *K. pneumoniae* is particularly concerning, the true clinical significance of the other *Klebsiella* spp. has not yet been fully elucidated due to the species misidentification. This addresses the need to investigate the prevalence of MDR on species level. MDR is usually defined as acquired non-susceptibility to at least one agent in three or more classes of antimicrobial agents among those that are considered as potentially effective against wild-type pathogens (Magiorakos et al., 2012). Currently there is no definition of genotypically MDR; therefore, we defined it as the detection of at least one resistance gene in at least three of the antibiotic classes included in ResFinder (Table 2). A total of 94.0% of the isolates within the *K. pneumoniae* complex were found to be genotypically MDR, whereas the proportion of MDR isolates was low in both *K. oxytoca* (18.8%) and *K. michiganensis* (0.0%). We detected significantly fewer resistance genes in *K. oxytoca* compared to *K. pneumoniae* and *K. variicola* (*p* < 0.001, Figure 3A) but not any significant differences in the number of plasmids between species (all values of *p* > 0.05, Figure 6A). The most frequently predicted plasmid replicons among all species in our study were the IncFIB (60 plasmid replicons in 47 isolates) and IncFII (47 plasmid replicons in 40 isolates; Figure 5). The IncFIB and IncFII plasmids have previously been reported to be the predominant plasmids found in *Klebsiella* (Cao et al., 2014; Long et al., 2017a).

A relatively high discordance for antibiotic susceptibility results was observed between phenotypic AST and WGS-AST, regardless of the bioinformatics tool used. This is mainly due to the fewer cases of resistance observed by phenotypic AST compared to the predictions made by ResFinder and 1928 (Tables 3, 5), also when excluding the cases when disc diffusion testing was not performed. However, a principal limitation of genotypic methods is that detected genetic markers are not necessarily expressed and translated into phenotypic resistance. False resistance can thus be expected for genetic markers that are tightly regulated, such as efflux pumps or inducible β-lactamases (Ferreira et al., 2020). Thereof, the high rate false-resistant results observed for quinolone (Tables 3, 5) for example, is most likely due to that the WGS-AST detected the presence of the gene *oqxA*, encoding an efflux pump conferring resistance to fluoroquinolones. In contrast, a few cases were determined as resistant by phenotypic AST but not by ResFinder nor 1928. Possible explanations for this discordance include the occurrence of natural resistance not found genotypically or the presence of a currently undescribed allelic variant related to resistance.

The misidentification of isolates within the *K. pneumoniae* complex has led to the deposition of misidentified reference strains in public repositories, including the public *K. pneumoniae* MLST scheme. Therefore, the MLST scheme for *K. pneumoniae* has previously been applied for typing other species within the *K. pneumoniae* complex. This may limit the use of MLST as a typing tool for molecular epidemiology in *Klebsiella* spp. and may further contribute to the misidentification problems within the *K. pneumoniae* complex (Long et al., 2017a). Consequently, the *K. variicola* MLST was recently developed allowing for molecular epidemiological analysis of *K. variicola* isolates by assignation of ST (Barrios-Camacho et al., 2019).

The *Klebsiella* isolates were found to be highly diverse comprising of e.g., 35 distinct STs for *K. pneumoniae*, 17 for *K. variicola*, and 10 for *K. oxytoca*. Earlier studies have also reported high genetic diversity among clinical *Klebsiella* spp. isolates (Wang et al., 2013; Ejaz et al., 2017; Wyres et al., 2020). For example, Wyres et al. (2020) observed a high diversity in their study of clinical *Klebsiella* spp. isolates in South and Southeast Asia with ST14 (16.0%) and ST231 (17.0%) as the dominating STs for the *K. pneumoniae* isolates from South Asia sites (Wyres et al., 2020). The most common ST among the *K. pneumoniae* isolates in this study was ST14 (10.0%), which has been associated with clonal spread of resistant *K. pneumoniae* (Giske et al., 2012; Mshana et al., 2013; Moubareck et al., 2018; Wyres et al., 2020).

Although MinION does not suffer from PCR bias introduction (Shagin et al., 2017) as found in Illumina, it does have a higher proportion of errors due to poor quality and issues sequencing homopolymer regions (Istace et al., 2017). As such, MinION long-reads can detect antimicrobial resistance mediated by acquired resistance genes, but it does not provide enough accuracy to identify resistances from chromosomal point mutations (Judge et al., 2016). In our study, the MinION long-reads identified similar genotypic findings, e.g., the species identification with WIMP on MinION long-reads confirmed the results found from the analysis of Illumina short-reads with a few exceptions. The automated workflow, EPI2ME, on the MinION long-reads rapidly provided clinically relevant information (~6 h.) and required minimal bioinformatics know-how, which seems promising for nanopore-based sequencing for future applications in clinical microbiology.

## Conclusion

The obtained results emphasize the importance of high-resolution genomic methods for identification and characterization of *Klebsiella* spp. For 39% of the isolates reported as *K. pneumoniae* by routine microbiological methods, the species were corrected based on the results from the genotypic species identification by ANI. Moreover, a very high prevalence (94%) of genotypically MDR isolates was observed among the species of the *K. pneumoniae* complex. These results together indicate an underrecognized clinical importance of other members of the *K. pneumoniae* complex than *K. pneumoniae*.

## Data Availability Statement

The datasets presented in this study can be found in online repositories. The names of the repository/repositories and accession number(s) can be found at: https://www.ncbi.nlm.nih.gov/, PRJNA606666.

## Ethics Statement

The study was approved by the Regional Ethical Review Board of Gothenburg (376-11). As the present study only focused on bacterial isolates recovered from cultures included in the routine patient care, no individual written consent was needed.

## Author Contributions

The conception and design of the study were done by all authors. PS performed the bioinformatics analyses of Illumina short-read data. JB performed the experimental work and bioinformatics analyses related to the MinION long-read data. AT, HE, and MF supervised PS and JB. DT wrote the original draft of the manuscript, curated the data, and performed downstream sequence analyses using ANI, MLST, and 1928, statistical analyses, and visualizations. All authors contributed to the article and approved the submitted version.

### Conflict of Interest

FD was employed by the company 1928 Diagnostics. HE was employed by the company Unilabs AB.

The remaining authors declare that the research was conducted in the absence of any commercial or financial relationships that could be construed as a potential conflict of interest.

## Footnotes

^1^https://eucast.org/

^2^https://cge.cbs.dtu.dk/services/

^3^https://www.1928diagnostics.com/

^4^https://cge.cbs.dtu.dk/services/

^5^https://pubmlst.org

^6^https://mlstkv.insp.mx

## Abbreviations

ANI: Average nucleotide identity
ARMA: Antimicrobial resistance mapping application
AST: Antibiotic susceptibility testing
CGE: Center for genomic epidemiology
ESBL: Extended spectrum β-lactam
EUCAST: European committee on antimicrobial susceptibility testing
MALDI-TOF MS: Matrix-assisted laser desorption-time of flight mass spectrometry
MDR: Multidrug-resistance
MLST: Multilocus sequence typing
NCBI: National center for biotechnology information
PCR: Polymerase chain reaction
QUAST: Quality assessment tool for genome assemblies
SPAdes: St. Petersburg genome assembler
ST: Sequence type
WGS: Whole-genome sequencing
WIMP: What’s-In-My-Pot
